# Igniting autophagy through the regulation of phase separation

**DOI:** 10.1038/s41392-020-0154-6

**Published:** 2020-05-01

**Authors:** Shouheng Jin, Jun Cui

**Affiliations:** MOE Key Laboratory of Gene Function and Regulation, State Key Laboratory of Biocontrol, School of Life Sciences, Sun Yat-sen University, 510275 Guangzhou, Guangdong China

**Keywords:** Cell biology, Molecular biology

A recent paper published in *Nature* by Fujioka et al. demonstrated that the Atg1 (also known as ULK1 in mammals) complex undergoes phase separation to form a liquid-like biomolecular condensate, thereby promoting the formation of a preautophagosomal structure (PAS) to activate autophagy in yeast.^1^ This research discovered the details of a mechanism of phase separation underlying autophagy initiation and provided a better understanding of the biological condensates.

Liquid–liquid phase separation (LLPS) of biomolecules is a widespread cellular phenomenon that modulates the generation of biomolecular condensates. The distinguishing features of phase separation-induced biomolecular condensates are their liquid-like nature, which enable the rapid exchange of components. Phase separation mediates a range of physiological roles, such as accelerating specific biocherical reactions and isolating specific proteins.^2^ The study of LLPS has led to great progress in delineating the roles of macromolecules. Autophagosomes, the formation of which are mediated by a number of autophagy-related (ATG) proteins, are macromolecular complexes in eukaryotic cells. A PAS is a transient structure regulated by nutrient conditions that invariably forms on the vacuolar membrane upon starvation in yeast.^1^ The Atg1 complex, which consists of Atg1, Atg13, Atg17, Atg29 and Atg31, is essential for the initiation of PAS formation. After maturation, a PAS recruits downstream ATG proteins and vesicles to serve as a site for autophagosome formation.

To study whether the behavior of phase separation has a role in autophagy initiation, Fujioka et al. examined the PAS in yeast and observed that GFP-Atg13 formed puncta upon nitrogen depletion and that the puncta rapidly dissolved in the presence of a nitrogen source, suggesting that the PAS is a highly dynamic and temporal entity. The authors further employed fluorescence recovery after photobleaching (FRAP) and fluorescence-correlation microscopy (FCS) analysis and observed the exchange of Atg13 within the PAS. Specifically, the enlargement of one PAS punctum coincided with the reduction of another. The researchers further studied the LLPS process by reconstructing the Atg1 complex in vitro and found that the mixture of purified Atg1, Atg13 and Atg17-Atg29-Atg31 resulted in the immediate onset of phase separation. These ATG proteins colocalized together within droplets, and the phase separation was most efficient at pH 6.0, which is a suitable pH for PAS formation in the physiological environment of yeast. In addition, the author confirmed that Atg13 and Atg17-Atg29-Atg31 were scaffolds for the phase separation of the Atg1 complex, and Atg1 was the client for the formation of the condensates.

Under normal cellular conditions, Atg13 is highly phosphorylated by TORC1 to inhibit the formation of the Atg1 complex, while the phosphorylation of Atg13 is eliminated during the autophagy-induced process. To reveal the mechanism by which phase separation is finely regulated, Fujioka et al. isolated phosphorylated Atg13 that had been incubation with purified TORC1 protein and found that this phosphorylated Atg13 could no longer be separated from Atg17-Atg29-Atg31, indicating that the phosphorylation of Atg13 inhibited phase separation behavior. Previous studies indicated that Atg1 is activated by starvation and undergoes autophosphorylation. The authors found that the phase separation of Atg13 and Atg17-Atg29-Atg31 facilitated the activation of Atg1 kinase, promoting the subsequent autophosphorylation of Atg1 and the phosphorylation of Atg13 and Atg29. Moreover, the authors found that Atg1-mediated phosphorylation suppressed the phase separation of the Atg1 complex. Atg1-complex droplets dissolved within minutes in the presence of Atg1 kinase activity in vitro, the PASs remained for several hours despite their activation of Atg1 kinase in cells, suggesting a more subtle regulatory mechanism in vivo. Further studies indicated that the phosphatase Ptc2 can dephosphorylate the Atg1 complex and promote its phase separation. Hence, the authors demonstrated that the phase separation of the Atg1 complex is regulated by phosphorylation and dephosphorylation.

Upon autophagy activation, the autophagy-related vacuolar membrane protein Vac8 interacts directly with Atg13 to tether PAS to the vacuolar membrane. Using giant unilamellar vesicles (GUVs), the authors also investigated whether Atg1-complex droplets could be tethered to the membranes through Vac8 in vitro and found that the Atg1 complex was localized and anchored to the GUVs with wild-type Vac8 but not to the GUVs with a Vac8 mutant. The authors also showed that the PASs localized to GUVs fused with each other to form large droplets, indicating that the droplets remained in a liquid-like state in the GUVs. Further research showed that, through a specific protein-protein interaction, an early PAS is a liquid-like condensate that is tethered to the vacuolar membrane.

**Fig. 1:**
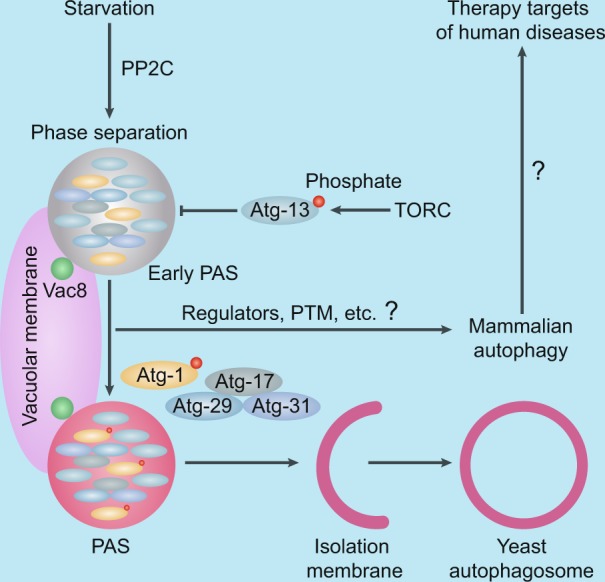
Phase separation ignites autophagy. Under autophagy-inducible conditions, decreased TORC1 activity promotes the dephosphorylation of Atg13 by PP2C, leading to the formation of the Atg1 complex. The Atg1 complex then undergoes phase separation to generate an early PAS, which is tethered to the vacuolar membrane via Vac8. The liquid droplet induces the autophosphorylation of the Atg1 kinase, which subsequently recruits downstream Atg proteins, thus prompting the formation of autophagosomes

Using a train of elegant experiments, Fujioka et al. reconstructed early PASs in vitro and confirmed that the Atg1 complex exhibits phase separation behavior, which is strictly regulated by intracellular pH value and phosphorylation modification. The researchers demonstrated that an early PAS was generated from the phase separation of the Atg1 complex and was localized to the vacuolar membrane through its association with Vac8. The early events in autophagy were discerned in this study of biomolecular PAS condensates (Fig. 1). Recently, rapid progress has been made in the identification of the roles of phase separation in selective autophagy degradation. mTOR regulates LLPS-mediated autophagic degradation of PGL-1/-3.^3^ p62 recognizes the polyubiquitination chains on the target protein and initiates p62 polymerization to induce phase separation, thus driving autophagic cargo concentration and segregation.^4^ In addition, selective autophagic cargo aminopeptidase I (Ape1) undergoes phase separation to form semiliquid droplets, which is important for the subsequent selective sequestration by Atg19 and Atg8-PE in yeast.^5^ However, the molecular mechanisms of phase separation in autophagy activation and regulation remain poorly understood. Fujioka et al. showed that a PAS emerges from the phase separation of ATG proteins, which provides insight into the origin of the double membrane of autophagosomes. Although autophagy is modulated by a set of posttranslational modifications (PTMs), it remains unknown whether other PTMs, such as ubiquitination, reversibly affect the phase separation of ATG proteins that initiate autophagy. Deficiency of autophagy may lead to human disease, especially neurodegenerative and inflammatory disorders and cancer. Several recent studies indicate that p62 condensates are involved in autophagic cargo concentration and segregation and triggers the antioxidant cell response,^2,4^ while the investigation of phase separation-directed autophagy in human diseases has not yet been reported. Phase separation-mediated autophagy may have essential physiological roles. The dissection of the underlying mechanism can provide a theoretical basis and therapeutic targets for the treatment of human diseases.
